# Transcriptome Analysis of Apple Leaves with Apple Necrotic Mosaic Virus-Associated Mosaic Symptoms

**DOI:** 10.3390/plants14121787

**Published:** 2025-06-11

**Authors:** Dehang Gao, Fei Xing, Qin Yan, Zhixiang Zhang, Binhui Zhan, Meiguang Lu, Yunlong Ma, Hongqing Wang, Shifang Li, Jipeng Xie

**Affiliations:** 1 Department of Fruit Science, College of Horticulture, China Agricultural University, Beijing 100193, China; 2 State Key Laboratory for Biology of Plant Diseases and Insect Pests, Institute of Plant Protection, Chinese Academy of Agricultural Sciences, Beijing 100193, China; 3 National Citrus Engineering Research Center, Citrus Research Institute, Southwest University, Chongqing 400712, China; 4 Department of Plant Pathology, China Agricultural University, Beijing 100193, China; 5 Center for Biosafety, Chinese Academy of Inspection and Quarantine, Sanya 572024, China

**Keywords:** apple mosaic disease, ApNMV, transcriptome, ROS

## Abstract

Apple mosaic disease (AMD) is a widespread viral disease affecting apple-growing regions around the world. Recent studies have identified a novel ilarvirus, apple necrotic mosaic virus (ApNMV), as the major causal agent of AMD in China. However, the molecular mechanisms underlying AMD pathogenesis and the global gene expression changes during mosaic symptom development remain largely unknown. In this study, we performed transcriptome analysis to investigate apple gene responses to AMD. A total of 815 differentially expressed genes (DEGs) were identified in mosaic leaves compared to healthy controls, while 1050 DEGs were found between symptomless leaves (infected with ApNMV) and mosaic leaves. Functional enrichment analysis revealed that these DEGs were predominantly involved in carbohydrate metabolism, oxidation-reduction processes, secondary metabolite biosynthesis, and plant hormone signal transduction. Further biological assays demonstrated that the manifestation of mosaic symptoms in apple leaves was associated with reactive oxygen species (ROS) accumulation and downregulation of ROS-scavenging genes. Collectively, our findings provide new insights into the molecular basis of ApNMV-induced mosaic symptom development in apple and offer potential targets for the management of AMD.

## 1. Introduction

Apple (Malus domestica) is a globally important fruit crop known for its high nutritional value and economic significance. However, apple trees are susceptible to infection by at least 19 viruses and virus-like pathogens, including apple stem pitting virus, apple stem grooving virus (ASGV), apple chlorotic leaf spot virus (ACLSV), apple mosaic virus (ApMV), apple necrotic mosaic virus (ApNMV), and viroids such as apple scar skin viroid [1]. Studies have shown that ASGV infection caused a 12–30% yield reduction in the ‘Golden Delicious’ apple cultivar, and the average height and diameter of cultivars were 23.4% lower and 13.7% smaller than healthy apple trees [2]. Moreover, apple mosaic disease (AMD) caused by either ApMV or ApNMV represents a significant threat to apple production due to its detrimental effects on leaf photosynthesis [3].

AMD was first reported in Europe and was confirmed to be a graft- and bud-transmissible viral disease [4,5,6,7]. Infected leaves typically exhibit pale-yellow chlorotic spots, amorphous chlorotic bands along or between veins, and characteristic mosaic patterns [7,8]. For decades, ApMV was considered the sole causal agent of AMD in major apple-producing regions. However, recent studies have failed to detect ApMV in apple trees displaying mosaic symptoms in China [9]. Instead, a novel ilarvirus named apple necrotic mosaic virus (ApNMV), closely related to prunus necrotic ringspot virus (PNRSV) and ApMV, was identified through next-generation sequencing and was shown to be strongly associated with AMD cases in China and other apple-producing countries [9,10,11,12]. ApNMV was detected at high rates of 82.6% and 92.1% in apple leaves showing mosaic symptoms in 2017 and 2018, respectively [9,10].

Like ApMV and PNRSV, ApNMV belongs to subgroup 3 of the genus *Ilarvirus* within the family *Bromoviridae* [9]. The ApNMV genome consists of three positive-sense single-stranded RNAs (RNA1, RNA2, and RNA3) and a subgenomic RNA4 derived from RNA3 [9]. RNA1 encodes the 1a protein, which contains conserved methyltransferase and helicase domains. The 1a protein is capable of self-interaction and also interacts with the RNA-dependent RNA polymerase (2a^Pol^) encoded by RNA2 [13]. RNA3 encodes the movement protein (MP), while the coat protein (CP), involved in the long-distance movement of the virus, is encoded by the subgenomic RNA4 [9,13].

As obligate intracellular parasites, viruses rely on intricate molecular interactions with host plants to establish infection. Previous studies have identified two key mechanisms underlying ApNMV–host interactions [14,15]. One mechanism involves MdBT2, a nitrate-responsive BTB/TAZ domain-containing protein, which promotes the ubiquitination and degradation of the ApNMV 1a protein and disrupts the 1a–2a^Pol^ interaction, thereby restricting viral replication [14]. Another mechanism involves the suppression of mitochondrial ATP synthase oligomycin sensitivity-conferring protein (OSCP) transcription by ApNMV in an ABI5-dependent manner to facilitate viral infection [15]. However, the molecular basis of mosaic symptom development during ApNMV infection remains unclear.

Mosaic is one of the most common symptoms in virus-infected plants [16]. Accumulating evidence suggests that mosaic symptom development is closely associated with the accumulation of reactive oxygen species (ROS) [17,18,19]. Viral infections trigger a rapid burst of ROS, including hydroxyl radicals (•OH), superoxide anions (O_2_^•−^), singlet oxygen (^1^O_2_), and hydrogen peroxide (H_2_O_2_) [20]. Excessive ROS can cause oxidative damage to lipids, DNA, and proteins, leading to cellular dysfunction and the formation of mosaic symptoms [21,22]. However, whether ROS or other host factors contribute to the development of ApNMV-associated mosaic symptoms remains to be determined.

Our study aims to elucidate the molecular basis of mosaic symptom formation in apple leaves infected with ApNMV via transcriptome analysis and experimental validation. We performed RNA-sequencing analysis on three groups of apple leaves including (1) no mosaic symptoms but ApNMV-positive leaves (designated as NMA), (2) mosaic and ApNMV-positive leaves (designated as MA), and (3) healthy controls (virus-free and symptomless). Transcriptomic analysis and functional verification revealed multiple signaling pathways including ROS- and plant hormones-related pathways might be involved in the development of mosaic symptoms. Ultimately, our findings provide a comprehensive transcriptomic overview of ApNMV-infected apple leaves and highlight a potential role of ROS in the development of AMD symptoms.

## 2. Results

### 2.1. Confirmation of the Presence of ApNMV in Mosaic and Asymptomatic Apple Leaves

Previous studies have demonstrated a strong association between ApNMV and the mosaic disease symptoms observed in apple trees in China [10]. To investigate the molecular mechanisms underlying mosaic symptom development in apple leaves, we collected branches showing typical mosaic symptoms from orchard-grown apple trees. These symptomatic branches were used as scions and grafted onto virus-free apple seedlings (used as rootstocks). Approximately one month after grafting, some of the newly emerged leaves on the rootstocks exhibited mosaic symptoms, while others remained asymptomatic.

**Figure 1:**
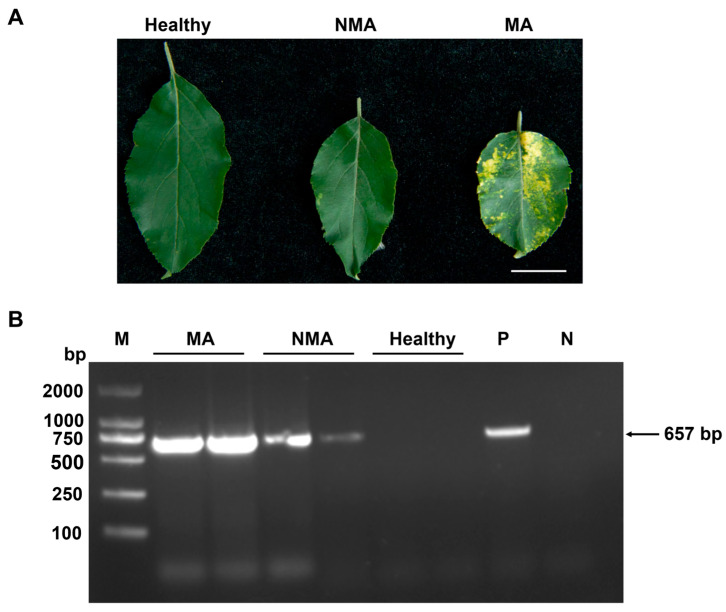
Detection of ApNMV in newly emerged leaves from grafted apple seedlings. (**A**) Apple leaves with healthy, NMA, and MA. MA: leaves exhibiting mosaic symptoms and testing positive for ApNMV; NMA: no mosaic symptoms but positive for ApNMV; Healthy: virus-free apple seedlings. Scale bar represents 2 cm. (**B**) Reverse transcription PCR (RT-PCR) was used to detect ApNMV genomic RNA in newly emerged symptomatic and asymptomatic leaves, as well as in virus-free apple seedlings. The arrow indicates the specific band corresponding to the ApNMV target fragment. M: DNA marker; P: positive control using plasmid containing ApNMV RNA3 (encoding CP) served as DNA template; N: negative control with ddH_2_O as template.

Reverse transcription PCR (RT-PCR) was performed to detect the presence of ApNMV genomic RNA in both NMA and MA leaves. The results confirmed that ApNMV was present in both leaf types, whereas no ApNMV was detected in the virus-free control seedlings (Figure 1). Further, reverse transcription-quantitative PCR (RT-qPCR) analysis demonstrated that the viral accumulation level of ApNMV in MA leaves was strikingly higher than that in NMA samples (Supplemental Figure S1). Total RNA was extracted from each group, and mRNA was prepared from four biological replicates per group. These samples were then subjected to high-throughput RNA sequencing (RNA-seq) for transcriptome analysis.

These results indicated that the apple samples for RNA-seq were reliable.

### 2.2. Overview of RNA-Seq Data

To profile gene expression changes during ApNMV-induced mosaic symptom development in apple leaves, RNA-seq libraries were constructed for 12 samples, including four biological replicates from each group: healthy control, NMA, and MA. A total of 94.48 GB of data, including 629,957,058 raw reads, were obtained from the 12 libraries. The number of reads ranged from 52,921,004 to 54,835,040 for the healthy control, from 49,028,714 to 53,209,290 for NMA, and from 51,285,302 to 54,162,150 for MA. After adapter trimming, low-quality base filtration, and removal of short reads, 73.7 GB of high-quality data, comprising 491,401,666 clean reads, were obtained, corresponding to a retention rate of 78.03%. The clean reads for each group ranged from 37,746,518 to 43,350,980 for the healthy control, from 39,154,772 to 40,655,122 for NMA, and from 39,984,106 to 43,130,050 for MA, covering 89.71%, 88.02%, and 88.16% of the apple genome, respectively. Furthermore, the percentage of reads uniquely mapped to the apple genome was over 63.18%, 59.88%, and 61.51%. For each library, more than 99.98% of the clean reads had quality scores at Q20, and 98.94% had scores at Q30. The GC content for these libraries ranged from 46% to 48%. These results indicated that the RNA-seq data was abundant and reliable. A summary of the transcriptome data is presented in Supplemental Table S1.

### 2.3. Analysis of DEGs in Apple Leaves with Healthy, NMA, and MA

**Figure 2:**
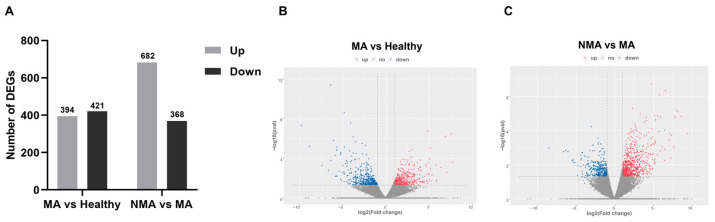
Volcano plots illustrating the differentially expressed genes (DEGs) in the MA vs. healthy and NMA vs. MA comparisons. (**A**) Total number of significantly up- and downregulated genes from the transcriptome data. (**B**,**C**) Volcano plots illustrating the DEGs in the MA vs. healthy and NMA vs. MA comparisons. Red and blue colors represent upregulated and downregulated genes, respectively.

**Figure 3:**
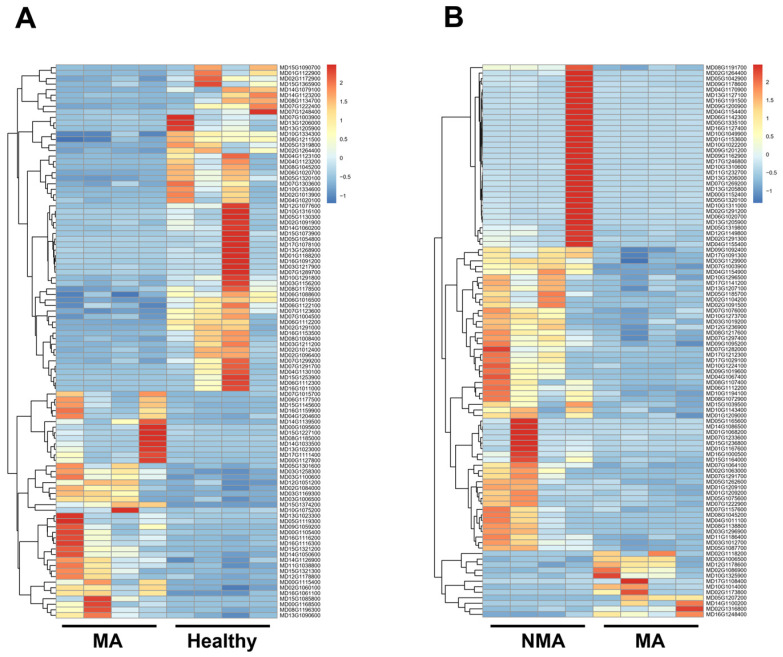
Hierarchical clustering of DEGs in apple leaves from healthy vs. MA (**A**) and NMA vs. MA (**B**) groups based on log2 (fold change) FPKM values. The color scale (from blue to red) indicates the intensity of gene expression.

To identify apple genes significantly altered during ApNMV-associated mosaic symptom development, differentially expressed genes (DEGs) were analyzed from the transcriptome profiles of the 12 libraries using thresholds of false discovery rate (FDR)-adjusted *p* < 0.05 and log2 (Fold Change) > 1 or log2 (Fold Change) < −1, which were considered significantly enriched for multiple comparisons. Comparative analysis revealed a total of 815 DEGs between the healthy and MA groups, consisting of 394 (48.34%) upregulated and 421 (51.66%) downregulated genes (Figure 2A,B). Additionally, 1050 DEGs were identified between the NMA and MA groups, with 682 (64.95%) upregulated and 368 (35.05%) downregulated genes (Figure 2A,C). The hierarchical clustering of these DEGs further highlighted distinct expression patterns for these comparisons (Figure 3). These data demonstrated the robust changes of apple genes in response to ApNMV infection.

### 2.4. Functional Enrichment Analysis of DEGs

**Figure 4:**
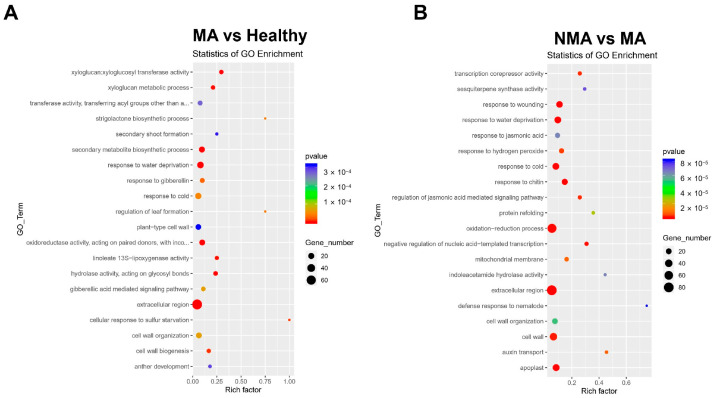
GO analysis of DEGs. (**A**,**B**) GO enrichment analysis of DEGs in the MA vs. Healthy and NMA vs. MA groups. The X-axis represents the number of DEGs, while the Y-axis shows the names of GO terms. The rich factor indicates the degree of enrichment of DEGs. The circle area corresponds to the number of enriched DEGs in each pathway. The rich factor reflects the degree of enrichment of DEGs, and the circle area indicates the number of enriched DEGs in each pathway. Note: The complete names of the third and twelfth columns in Figure 4A are “transferase activity, transferring acyl groups other than amino-acyl groups” and “oxidoreductase activity, acting on paired donors, with incorporation or reduction of molecular oxygen, NAD(P)H as one donor, and incorporation of one atom of oxygen”, respectively.

Gene Ontology (GO) analysis revealed that the DEGs identified between the healthy and MA groups were significantly enriched in secondary metabolite biosynthesis, response to water deprivation, and oxidoreductase activity (Figure 4A, Supplemental Figure S2). Notably, a substantial number of genes encoding proteins with extracellular regions, such as receptor-like protein kinase *FERONIA* (MD12G1190700) and *glutamate receptor 3.6-like* (MD15G1229500), which were enriched in both healthy vs. MA and NMA vs. MA groups (Figure 4B, Supplemental Figure S2). For the DEGs in the NMA vs. MA comparison, the biological processes significantly enriched included oxidation-reduction, response to wounding, and response to water deprivation (Figure 4B, Supplemental Figure S2).

**Figure 5:**
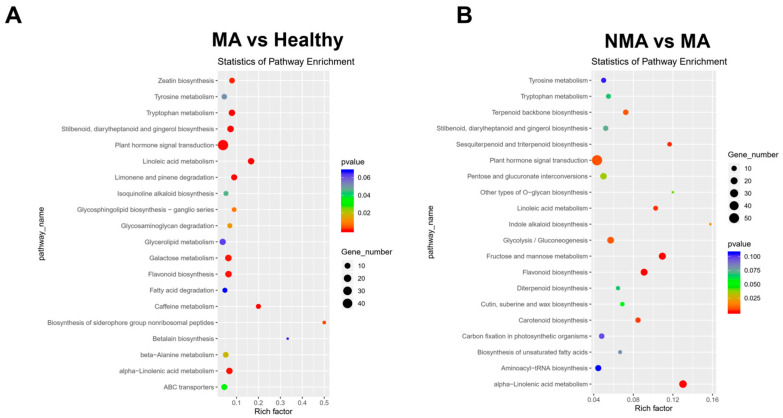
KEGG analysis of DEGs. (**A**,**B**) KEGG enrichment analysis of DEGs in the MA vs. Healthy and NMA vs. MA groups. The X-axis represents the number of DEGs in KEGG pathways, while the Y-axis displays the names of the pathways. The rich factor reflects the degree of enrichment of DEGs, and the circle area indicates the number of enriched DEGs in each pathway.

To gain deeper insights into the molecular and biological functions of the identified DEGs, the genes were mapped to the Kyoto Encyclopedia of Genes and Genomes (KEGG) database. The significant enrichment analysis indicated that DEGs in the MA vs. healthy comparison were primarily enriched in plant hormone signal transduction, a pattern also observed in the NMA vs. MA comparison (Figure 5). Additionally, in the MA vs. healthy groups, enriched pathways included flavonoid biosynthesis, galactose metabolism, tryptophan metabolism, stilbenoid, diarylheptanoid, and gingerol biosynthesis (Figure 5A). Meanwhile, the NMA vs. MA comparison revealed significant enrichment in pathways related to glycolysis/gluconeogenesis, fructose and mannose metabolism, flavonoid biosynthesis, and alpha-linolenic acid metabolism (Figure 5B). These results suggested that these biological processes might be affected by ApNMV infection.

### 2.5. Validation of the Transcriptome Data by RT-qPCR

**Figure 6:**
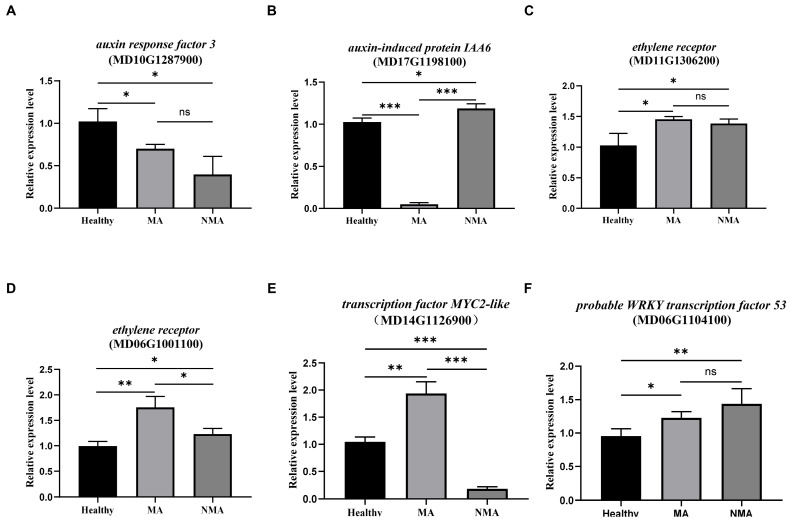
RT-qPCR was performed to validate the expression of genes selected from the transcriptome results. (**A**–**F**) Specific genes involving in various phytohormone pathways, along with *WRKY53*, were analyzed. The expression levels of each gene were normalized to an internal control *MdUBQ* (XM008360582). Asterisks indicate statistically significant differences (* *p* ≤ 0.05; ** *p* ≤ 0.01; *** *p* ≤ 0.001; ‘ns’ indicates no significant difference), as determined by a two-tailed Student’s *t*-test. Error bars represent the standard deviation (SD) (n ≥ 3 for each treatment).

To validate the RNA-seq data in this study, the expression levels of six DEGs from healthy, NMA, and MA samples were selected for RT-qPCR analysis using specific primers (Supplemental Table S2). The selected DEGs included two genes involved in the auxin pathway: *auxin response factor 3* (MD10G1287900) and *auxin-induced protein IAA6* (MD17G1198100), two *ethylene receptor* genes (MD11G1306200, MD06G1001100), *MYC2-like transcription factor* (MD14G1126900), and *WRKY53* (MD06G1104100). Compared to healthy controls, the expression levels of two *ethylene receptor* genes, *MYC2-like transcription factor*, and *WRKY53* were significantly upregulated in MA samples, whereas *auxin response factor 3* and *IAA6* were downregulated (Figure 6). When comparing gene expression levels in MA to that in NMA, *ethylene receptor* (MD06G1001100) and *MYC2-like transcription factor* were increased, IAA6 was dramatically decreased, while *auxin response factor 3*, *ethylene receptor* genes (MD11G1306200), and *WRKY53* were unchanged (Figure 6). Collectively, the RT-qPCR data were largely consistent with the changes in gene expression observed in the transcriptome profile (Supplemental Table S3), confirming the reliability of the RNA-seq data.

### 2.6. Mosaic Symptoms Development Is Correlated with Down-Regulation of Peroxidases and ROS Production in Apple Leaves

**Figure 7:**
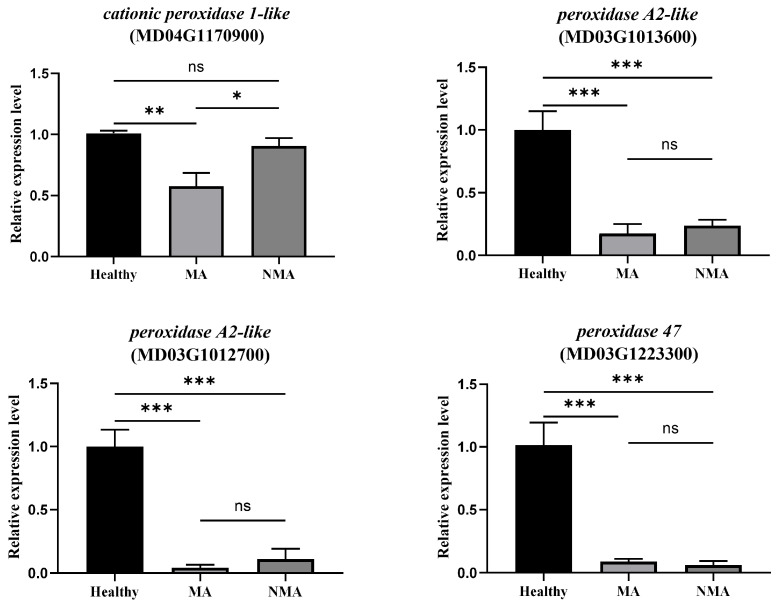
Quantification of *peroxidase* expression involved in ROS scavenging. The relative expression levels of peroxidases (ROS scavenging genes) in apple were analyzed by RT-qPCR. The expression levels of each gene were normalized to the internal control *MdUBQ*. Asterisks indicate significant differences (* *p* ≤ 0.05; ** *p* ≤ 0.01; *** *p* ≤ 0.001; ‘ns’ indicates no significant difference), determined by a two-tailed Student’s *t*-test. Error bars represent standard deviation (SD) (n ≥ 3 for each treatment).

Previous studies have demonstrated the involvement of ROS in the development of mosaic symptoms during compatible plant-virus interactions [17,18,19]. ROS production is usually accompanied by the downregulation of antioxidant enzymes or ROS scavenging pathways [23,24]. To investigate whether genes involved in ROS scavenging pathways are altered in mosaic apple leaves, we analyzed the transcriptome data. We found that the expression levels of various homologs of *peroxidase* including *cationic peroxidase 1-like* (MD04G1170900), *peroxidase A2-like* (MD03G1013600), *peroxidase A2-like* (MD03G1012700), and *peroxidase 47* (MD03G1223300), were downregulated in the MA group compared to the healthy control. Further RT-qPCR analysis confirmed that the expression levels of these four *peroxidase* homologs were significantly reduced in both the MA and NMA groups compared to the healthy control (Figure 7). In situ histochemical staining of H_2_O_2_ using 3,3′-diaminobenzidine (DAB) was performed to detect ROS in MA apple leaves. Clear and large brown spots, indicative of H_2_O_2_ production, were observed in leaf areas exhibiting mosaic symptoms (Supplemental Figure S3). These findings suggested that the development of ApNMV-associated mosaic symptoms in apple leaves is correlated with the downregulation of *peroxidases* and the accumulation of ROS.

## 3. Discussion

AMD poses a significant threat to the apple industry, causing economic losses worldwide. While the novel ilarvirus ApNMV has been identified as the major causal agent of mosaic diseases in apple trees in China [9,10], the molecular mechanisms underlying ApNMV-induced symptom development remain poorly understood. In this study, we employed high-throughput RNA-seq technology to analyze transcriptome changes during the development of mosaic symptoms. Our data revealed dysregulated primary and secondary metabolic pathways and ROS production in response to AMD.

ROS functions as a double-edged sword in plant–pathogen interactions. At low levels, it serves as a signaling molecule, while excessive ROS levels lead to oxidative stress [21,22]. Several studies have demonstrated that ROS is involved in symptom development during compatible plant–virus interactions [17,18,19,25]. For example, ROS bursts contribute to the development of mosaic and yellowing symptoms caused by cucumber mosaic virus and zucchini yellow mosaic virus [25]. Accumulation of H_2_O_2_ was observed in bamboo mosaic virus (BaMV)-induced chlorotic spots, with symptom severity enhanced by exogenous H_2_O_2_ application [18]. Consistent with these findings, histochemical analysis of ApNMV-infected apple leaves suggests that ROS accumulation plays a role in the development of mosaic symptoms. This was further supported by the downregulation of ROS scavenging-related genes, such as *peroxidases*, in MA and NMA samples compared to healthy controls.

ROS can be produced in various subcellular compartments, such as chloroplasts, mitochondria, peroxisomes, endoplasmic reticulum, and plasma membrane [21]. A recent study showed that potyvirus infections promote the accumulation of mitochondrial ROS (mROS) and malate, leading to mosaic symptoms in maize leaves [19]. It would be interesting to investigate whether the H_2_O_2_ produced during ApNMV infection originates from chloroplasts, mitochondria, or other subcellular compartments.

Phytohormones such as salicylic acid (SA), jasmonic acid (JA), and ethylene (ET) have long been recognized for their roles in plant defense against biotic and abiotic stresses [26]. They also play critical roles in plant–virus interactions. The biosynthesis and signaling pathways of these phytohormones influence virus infection and are themselves modulated by plant viruses, contributing to symptom development [27,28]. For instance, silencing *CORONATINE-INSENSITIVE 1* (COI1), the receptor for JA, accelerates symptom development and viral accumulation at the early stages of infection for potato virus Y and potato virus X [29,30]. The ET pathway is involved in the development of mosaic symptoms caused by cauliflower mosaic virus and cucumber mosaic virus. Virus-induced aberrant phenotypes, such as stunting and leaf curling, are similar to those seen in mutants compromised in auxin biosynthesis or signaling [31,32]. The replicase of tobacco mosaic virus interacts with *Arabidopsis* INDOLE-3-ACETIC ACID INDUCIBLE 26 (IAA26), IAA18, and IAA27 to induce stunting symptoms [33,34]. The differential regulation of marker genes in the JA, ET, and auxin pathways at both the pre-symptomatic (NMA) and symptomatic (MA) stages suggests a complex interplay of hormone networks in the manifestation of ApNMV-associated mosaic symptoms. Future studies investigating the roles of JA, ET, and auxin, as well as their cross-talk, will help to elucidate the underlying mechanisms of mosaic symptom development in apple leaves.

Transcription factors (TFs), which contain DNA-binding domains, play crucial roles in regulating the transcription of target genes by interacting with their *cis*-regulatory elements. Many plant TF families, including WRKY, NAC, MYB, bZIP, bHLH, and AP2/ERF, have been shown to participate in plant responses to various viruses [35]. In our study, TFs such as WRKY, NAC, MYB, bZIP, and bHLH were differentially expressed in ApNMV-infected apple leaves, suggesting their potential roles in ApNMV infection and mosaic symptom development. Notably, a large number of *WRKY* genes were dysregulated in the transcriptome data, with *WRKY53* (MD06G1104100) being significantly upregulated in both MA and NMA apple leaves compared to healthy controls. Previous studies have demonstrated that WRKY TFs can either restrict or promote virus infection. For example, NbWRKY1 and NbWRKY40 confer resistance to mulberry mosaic dwarf-associated virus and tomato mosaic virus, respectively [36,37], while knockdown of *WRKY41* or *WRKY54* in the Zheza-301 tomato cultivar reduces the accumulation of tomato yellow leaf curl virus DNA [38]. The roles of these TFs in ApNMV infection and symptom development warrant further investigation.

In conclusion, our study provides the first comprehensive transcriptome profile of apple genes in ApNMV-associated mosaic apple leaves, highlighting the involvement of multiple signaling pathways in mosaic symptom development. Moreover, the manifestation of mosaic symptoms is accompanied by ROS production and a decline in ROS scavenging genes. These findings offer valuable insights into the mechanisms underlying mosaic disease in apple trees and identify potential candidate genes that could be targeted for future AMD control. However, further functional validation of the involvement of these potential candidates in ApNMV infection and development of AMD is worthy of investigation.

## 4. Materials and Methods

### 4.1. Plant Materials for RNA-Seq

Apple branches whose leaves showed mosaic symptoms were collected in orchards, followed by grafting onto virus-free *Malus domestica* ‘Fuji’ apple seedlings (as rootstocks). Mosaic symptoms in newly emerged leaves of rootstocks appeared about 1 month post-grafting. The ApNMV-infected newly emerged leaves with mosaic symptoms and symptomless in the same rootstocks were harvested as MA and NMA samples, respectively. The leaf samples taken from virus-free seedlings served as healthy controls. These samples were harvested at the same timepoint, and subjected to high-throughput sequencing. Four biological replicates were obtained for each case.

### 4.2. RNA Extraction, Library Construction and Sequencing

The total RNA of apple leaves was extracted using Trizol reagent (Invitrogen, Carlsbad, CA, USA) following the manufacturer’s procedure. The quantity and purity were analyzed by Bioanalyzer 2100 and RNA 6000 Nano Lab Chip Kit (Agilent Technologies, Santa Clara, CA, USA) with RIN number >7.0. Approximately 3 μg of total RNA per sample was subjected to isolate Poly (A) mRNA with poly-T oligo attached magnetic beads (Invitrogen, Carlsbad, CA, USA). Following purification, the mRNA is fragmented into small pieces using divalent cations under elevated temperatures. Then the cleaved RNA fragments were reverse-transcribed to create the final cDNA library in accordance with the protocol for the mRNA Seq sample preparation kit (Illumina, San Diego, CA, USA), the average insert size for the paired-end libraries was 300 bp (±50 bp). Then paired-end sequencing was performed on an Illumina Hiseq^TM^ 4000 platform (LC Sciences, Hangzhou, China) according to the manufacturer’s instructions.

### 4.3. Quality Control

For the quality control of sequencing data, the raw data (raw reads) in fastq format were first processed by internal scripts. That is, clean data/reads were obtained by removing reads containing adapter, poly-N, or low-quality reads from raw data. The Q20, Q30, GC-content, and sequence duplication levels of clean data were calculated. High-quality clean data was used for subsequent analysis.

### 4.4. RNA-Seq Reads Mapping to Reference Genome

Alignment of reads to apple reference genome (ftp://ftp.bioinfo.wsu.edu/species/Malus_x_domestica/Malus_x_domestica-genome_GDDH13_v1.1/) using HISAT package (accessed on 10 February 2025), HISAT allows multiple alignments per read (up to 20 by default) and a maximum of two mismatches when mapping reads to the reference genome.

### 4.5. Analysis of Differential Gene Expression, GO Term and KEGG Pathway Enrichment

The mapped reads of each sample were assembled using StringTie. StringTie and edgeR were used to estimate the expression levels of all transcripts by calculating per kilobase of transcript sequence per millions of base pairs sequenced (FPKM). The differentially expressed genes were selected by log2 (fold change) >1 or log2 (fold change) <−1 and with statistical significance (*p* value < 0.05) by R package. Gene Ontology (GO; http://www.geneontology.org) and KEGG (http://www.genome.jp/kegg) enrichment analysis was performed using REVIGO (version 1.8.1) and KOBAS (version 3.0) software, respectively. These data were accessed on 10 February 2025.

### 4.6. RT-qPCR Analysis of DEGs

To validate the transcriptome data, several DEGs were selected for RT-qPCR assay with the RNA samples that were used for sequencing library construction. In detail, total RNA was extracted from apple leaves using RNAprep Pure Plant Plus Kit (TIANGEN BIOTECH (BEIJING), Beijing, China) according to the manufacturer’s protocol. RNA concentration and quality were assessed using a Nano-300 spectrophotometer (Allsheng Instruments, Zhejiang, China). First-strand cDNA synthesis was performed using 1 µg of total RNA and a HiScript II Q RT SuperMix for qPCR (+gDNA wiper) kit (Vazyme, Nanjing, China). RT-qPCR (volume of 10 µL) was carried out by adding 1 µL of cDNA, 0.5 µL of specific primers (10 µM) as the template into 2×Taq Pro Universal SYBR qPCR Master Mix (Vazyme, Nanjing, China) on a QuantStudio6 real-time PCR instrument (Applied Biosystems Inc., Foster City, CA, USA). Expression levels were normalized using *MdUBQ* (XM008360582) as the reference gene for apple. The 2^−ΔΔCT^ method was used for relative quantification. All experiments were performed with at least three biological replicates, and primers for RT-qPCR are listed in Table S2.

### 4.7. DAB Staining

Staining of mosaic apple leaves with DAB was performed as a method previously described [39]. The detached leaves were immersed in a 1 mg/mL DAB solution (TIANGEN BIOTECH (BEIJING), Beijing, China) and vacuum-infiltrated for 2 h, followed by overnight incubation at room temperature for staining. After staining, the leaves were placed in 95% (*v*/*v*) ethanol, decolorized in a boiling water bath, and then photographed.

## 5. Conclusions

In this study, we performed comprehensive transcriptome profiling of apple leaves exhibiting mosaic symptoms positive for ApNMV infection. Our results revealed that mosaic symptom development is closely linked to the accumulation of ROS and the downregulation of ROS-scavenging *peroxidase* genes. Functional enrichment analysis further demonstrated that numerous genes involved in secondary metabolism, oxidation-reduction processes, plant hormone signaling and encoding proteins with extracellular regions are significantly enriched during mosaic symptoms development. Additionally, some key TFs and plant hormone-related genes were differentially expressed at both asymptomatic and symptomatic stages, suggesting complex regulatory networks underpinning mosaic symptoms manifestation. These findings provide novel insights into the molecular mechanisms of ApNMV-associated mosaic symptoms and identify potential targets for future functional validation and molecular breeding of AMD-resistant apple cultivars.

## Data Availability

The raw sequence data reported in this paper have been deposited in the NCBI bioproject database with accession number PRJNA1233932, which is available at https://www.ncbi.nlm.nih.gov/bioproject/PRJNA1233932. The data was accessed on 11 March 2025.

## Supplementary Materials

The following supporting information can be downloaded at: https://www.mdpi.com/article/10.3390/plants14121787/s1, Supplemental Figure S1. Reverse transcription-quantitative PCR (RT-qPCR) analysis of accumulation level of ApNMV RNA3 in NMA and MA samples. Supplemental Figure S2. GO enrichment analysis of DEGs in apple leaves with healthy, MA, or NMA. Supplemental Figure S3. H_2_O_2_ production on mosaic apple leaves. Supplemental Table S1. Summary of RNA-seq data for apple leaves in this study. Supplemental Table S2. Primers for RT-qPCR analysis. Supplemental Table S3. The expression levels of 10 DEGs were selected from transcriptome data.
