# Supplementary material for: Transcriptome Analysis of Apple Leaves with Apple Necrotic Mosaic Virus-Associated Mosaic Symptoms
Source: Plants (Basel). 2025 Jun 11;14(12):1787. doi: 10.3390/plants14121787 (PMC12197047; doi:10.3390/plants14121787)
Supplement: Supplementary file 1 [file plants-14-01787-s001.zip › Supplemental figures and figure legends.pdf]

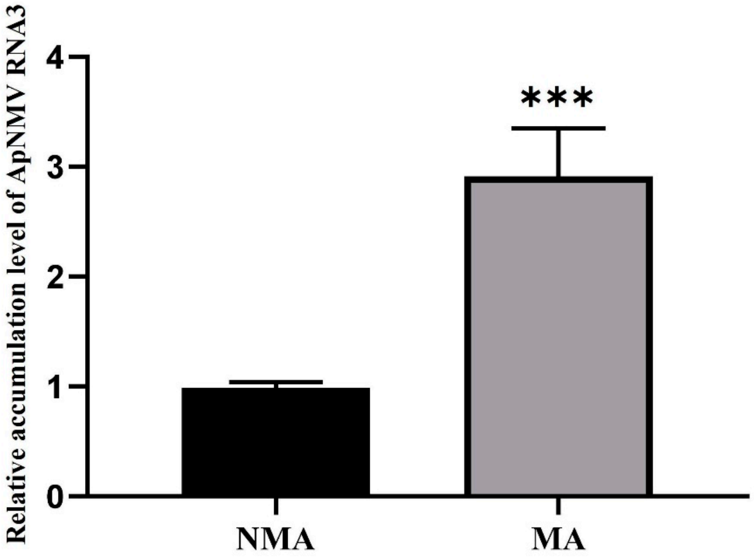

Supplemental Figure S1. Reverse transcription-quantitative PCR (RT-qPCR) analysis of accumulation level of ApNMV RNA3 in NMA and MA samples. Total RNA was extracted from NMA and MA apple leaves, and subjected to RT-qPCR assay. The viral accumulation level was normalized to an internal control *MdUBQ* (XM008360582). Asterisks indicate significant difference (***$P \leq 0.001$), analyzed by a two-tailed Student's *t*-test. Error bars represent standard deviation (SD) ($n \geq 3$ for each case).

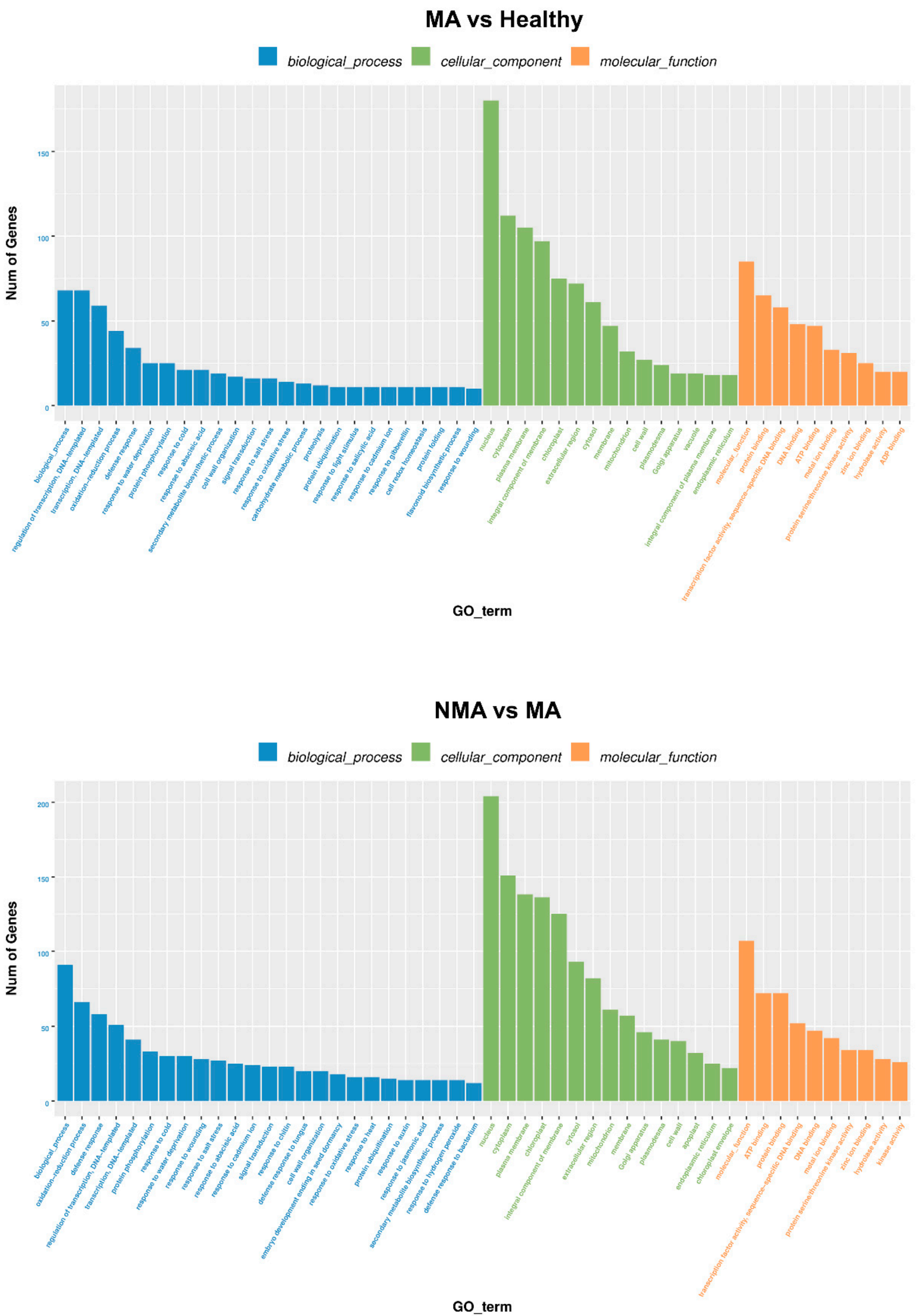

Supplemental Figure S2. GO enrichment analysis of DEGs in apple leaves with healthy, NMA, or MA. The X-axis corresponds to names of DEGs in GO terms. The Y-axis represents the numbers of DEGs. The genes were divided into three categories: biological process, cellular component, and molecular function.

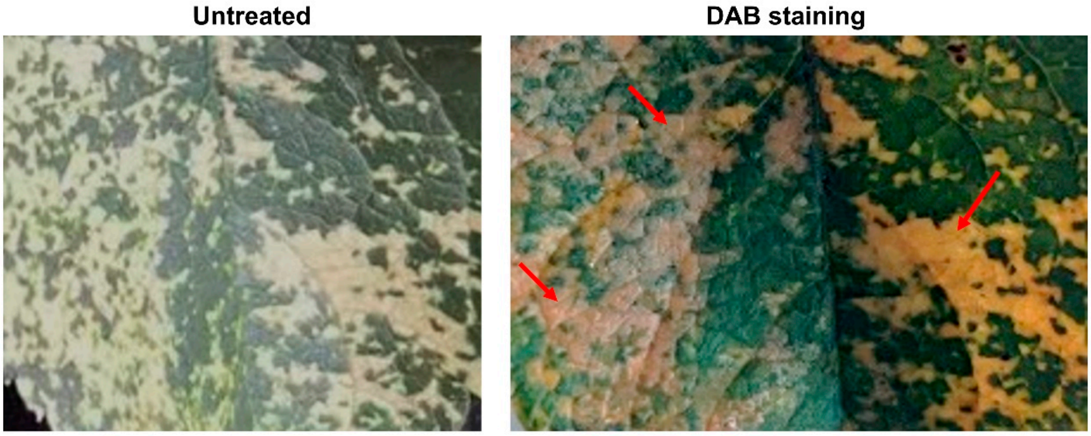

Supplemental Figure S3. H_2O_2 production on mosaic apple leaves. The ApNMV-infected mosaic apple leaves was stained by 3,3'-diaminobenzidine (DAB) to indicate H_2O_2 production. Red arrows indicate brown spots representing H_2O_2 accumulation.
